# Reconstruction of Iatrogenic Bile Duct Injuries following Laparoscopic Cholecystectomy

**DOI:** 10.12669/pjms.41.9.11870

**Published:** 2025-09

**Authors:** Ainul Hadi, Imranuddin Khan, Farrukh Ozair Shah

**Affiliations:** 1 Ainul Hadi Associate Professor, Surgical “A” Unit, Hayatabad Medical Complex, Peshawar, Pakistan; 2 Imranuddin Khan Specialist Registrar, Surgical “A” Unit, Hayatabad Medical Complex, Peshawar, Pakistan; 3 Farrukh Ozair ShahAssociate Professor, Surgical “A” Unit, Hayatabad Medical Complex, Peshawar, Pakistan

**Keywords:** Iatrogenic bile duct injury, Laparoscopic cholecystectomy, Roux-en-Y hepaticojejunostomy

## Abstract

**Objectives:**

To find out the management outcomes of iatrogenic bile duct injuries following laparoscopic cholecystectomy.

**Methodology:**

In this descriptive case series study total of 90 patients of both gender and who sustained extra hepatic biliary injuries, were included. Patients managed through ERCP and stenting and those with hepatobiliary malignancy were excluded. These 90 cases included those patients who sustained biliary injury during laparoscopic cholecystectomy at the department of surgery, Hayatabad Medical complex, Peshawar from July 2021 to June 2024 and those referred from peripheral hospitals and Afghanistan. Investigations modalities included abdominal ultrasonography and MRCP in all referred cases while ERCP was performed in selected cases. Procedures were performed by senior surgeons. Patients were followed up in outpatient department (OPD) for six months. Data was collected on a predesigned proforma and analyzed through SPSS version 16.

**Results:**

The age range of patients was 28-56 years with a mean age of 43 years (SD ± 2.5 years). Male to Female ratio was 1:5. The presenting complaints were pain (77.8%), jaundice (76.7%), fever (74.4%), biloma (50.0%) and persistent biliary leak (40.0%). Sites of injury involved CHD (26.7%), CBD (53.3%), porta hepatis (7.8%) and 12.2% cases had biliary strictures. The most commonly performed procedure was Roux- en-Y hepaticojejunostomy (54.4%) followed by Choledochodudenostomy (21.1%), primary repair (16.7%) and Roux-en-Y Portoenterostomy (7.8%). Postoperative morbidity included bile leakage (8.9%), wound infection (16.7%), recurrent cholangitis (20.0%) and anastomotic stenosis (3.3%). Mortality rate was 4.4% and overall success rate was 95.6%.

**Conclusions:**

CBD was the most frequent site injured during laparoscopic cholecystectomy. Roux-en-Y hepaticojejunostomy is the most commonly performed procedure with a good success rate in experienced hands.

## INTRODUCTION

According to literature, 10-15% of the adults are suffering from gall stones disease across the world.^1^ There is always associated risk of bile duct injury during open or laparoscopic cholecystectomy.^2^ The most commonly performed procedures for bile duct injuries are end to end anastomosis and Roux-en- Y biliary enteric anastomosis.^3^ The reported incidence of Iatrogenic bile duct injuries following open cholecystectomy is 0.1-0.3% and 2% for laparoscopic cholecystectomy.^4^ With increasing experience and availability of advanced laparoscopic instruments, the incidence of bile duct injuries has been dropped to less than 0.5%.^5^,^6^ Factors responsible for iatrogenic bile duct injuries are adhesions, presence of aberrant duct, excessive use of diathermy, unnecessary dissection near the Calot’s triangle and unexpected hemorrhage.^7^,^8^

Bile duct injury may be either complete or partial transection with resultant biliary leak, ligation of major bile duct and both of these, cause stricture formation in the long term. Iatrogenic bile duct injury reveals itself in the form of persistent drainage of bile in the drain, raised levels of serum bilirubin and alkaline phosphatase, localized or generalized peritonitis and signs of septicemia.^9^ Specific investigations to diagnose a case of biliary injury and plan subsequent management, include abdominal ultrasonography, CT scan (Computerized Tomograpghy Scan) abdomen, MRCP (Magnetic Resonance Cholangiopancreatography), ERCP (Endoscopic Retrograde Cholangiopancreatography) and intraoperative cholangiography.^9^-^12^ The purpose of this study was to find out the management outcomes of iatrogenic bile duct injuries following laparoscopic cholecystectomy in the department of general surgery.

## METHODOLOGY

This prospective study of 90 patients, was conducted at the department of surgery, Hayatabad Medical Complex (HMC) Peshawar, from July 2021 to June 2024. Initially 106 patients of either sex, sustaining iatrogenic injuries to extra hepatic biliary tree following laparoscopic cholecystectomy were included.

### Inclusion & Exclusion Criteria:

Eight patients did not complete their follow up visits and were excluded from the study. Five were managed through ERCP (Endoscopic Retrograde Cholangio Pancreatography) and stenting up with Hepatobiliary malignancies were also excluded. Ninety patients considered in study, were those cases who sustained iatrogenic bile duct injuries at the Department of Surgery, HMC and those referred from hospitals after biliary injury, by the gastroenterologists and three referred patients, picked in periphery and Afghanistan.

### Ethical Approval:

It was obtained from Institutional Ethical Review board (IRB No.: 1138; dated September 6, 2022).

Patients were thoroughly investigated by performing CBC (Complete Blood Count), LFTs (Liver Function Tests), PT (Prothombin Time), APTT (Activated Partial Thrombopastin Time), INR (International Normalized Ratio), Urea, Creatinine, blood sugar and serum electrolytes. HBsAg (Hepatitis B Screening), HCV (Hepatitis C Virus) and HIV (Human Immunodeficiency Virus) screening were also performed. Abdominal ultrasonography (U/S) and MRCP (Magnetic Resonance Cholangiopancreatography) were performed in all referred cases while ERCP was performed in selected cases to reach the final diagnosis. Procedures were performed under general anesthesia by experienced consultant surgeons through right subcostal incisions. Postoperatively, patients were strictly monitored in ward and HDU (High Dependency Unit).

Patients were followed in the outpatient department for six months. Follow up visits were scheduled at 10 days, one month, three months and six months after surgery. On first visit, LFTs and abdominal U/S were performed in all cases to assess liver function and look for postoperative collection. Wound was examined and stitches were removed. At 4th visit, MRCP was performed to check the patency of bilioenteric anastomosis in those cases who had dilated biliary tree or having one episode of ascending cholangitis. Data regarding age, sex, time elapsed between initial surgery and final diagnosis, surgical procedure and postoperative morbidity was recorded and analyzed through SPSS version 16.

## RESULTS

Ninety patients underwent laparoscopic cholecystectomy. Eight cases who failed to complete their follow up visits were excluded. Out of 90 patients, 15(16.7%) were males and 75(83.3%) were females with male to female ratio of 1:5 (MF = 1:5). The age range was from 28 to 56 years with a mean age of 43 years (SD ± 2.5 years). Seventeen (18.8%) patients had sustained bile duct injury in the department of Surgery, HMC Peshawar. Forty-one (45.6%) patients had been referred from different hospitals of KPK (Khyber Pukhtoon Khawa) while 8 (35.6%) out 90 cases had sustained bile duct injury during laparoscopic cholecystectomy in Afghanistan and then referred for the management of complications.

Seventeen (18.8%) patients who sustained bile duct in our surgical department, were managed in the same setting. Other patients referred from peripheral hospitals and Afghanistan, presented within 1.5 months following initial surgery. Their presenting complaints were pain in the right hypochondrium and epigastrium (n=70, 77.8%), jaundice (n=69, 76.7%), fever (n=67, 74.4%) and vomiting (n=58, 46.4%). In 45(50%) cases, there was sub-hepatic collection (biloma) and 36(40.0%) patients had persistent biliary leak through the drain placed during the initial surgery. Ten (11.1%) had developed biliary peritonitis. Eleven (12.3%) patients had biliary stricture which were picked up through ERCP and MRCP.

Diagnostic investigations performed, were abdominal ultrasonography in 73(81.1%) referred cases which revealed biloma in 45(50.0%) cases and free fluid in the peritoneal cavity in 10(11.1%) cases. ERCP was performed in 25(27.8%) cases. ERCP findings included Stricture 11(12.3%) and injury to the extra hepatic biliary tree in 14(15.6%) cases. MRCP was performed in all 73(81.1%) patients to define the anatomy of biliary tree and level of injury of 90 cases, 26.7% (n=24) had CHD (Common Hepatic Duct) injury, 53.3% (n=48) had CBD (Common Bile Duct) injury and 12.2% (n=11) had CBD strictures. 7.8% (n=7) patients had injury at the porta hepatis (Table-I).

**Table-I:** Site of Injury (n=90).

Site Involved	No of Cases operated in HMC	Peripheral hospitals	Afghanistan	Total (%)	Type of Injury
CHD Injury	6	10	8	24 (26.7)%	II
CBD Injury	11	21	16	48 (53.3%)	I
Biliary Strictures	0	7	4	11 (12.2%)	I,II
Porta Hepatis	0	3	4	7 (7.8%)	IV
Total	17 (18.8%)	41 45.6%)	32 (35.6%)	90 (100%)	

According to Bismuth classification, 53.3% (n=48) had Type-I injury, 26.7% (n=24) had Type-II injury and 12.3% (n=11) having biliary strictures, had both Type-I and II injuries. 7.8% (n=7) patients had Type-IV injury. Operative procedures included Roux-en-Y hepaticojejunostomy (n= 49, 54.4%), Choledochodudenostomy (n=19, 21.1%), Roux-en-Y Portoenterostomy (n=7, 7.8%) and primary anastomosis over a T-tube (n=15, 16.7%) (Table-II). Early postoperative complications included wound infection (n=15, 16.7%), bile leakage through drain (n=8, 8.9%) and acute episodes of recurrent cholangitis (n=18, 20.0%) which were treated conservatively with antibiotics. Late complications included stenosis (n-3, 3.3%). Two (2.2%) patient had CBD repair over T-tube and one (1.1%) had Roux-en Y hepaticojejunostomy. These patients undergone revision surgery (Table-III).

**Table-II:** Procedures Performed (n= 90).

Procedures	CHD	CBD	CBD Strictures	Porta Hepatis	Total	Percentage
Roux-en-Y Hepaticojejunostomy	23	20	6	0	49	54.4%
Choledochodudenostomy	0	14	5	0	19	21.1%
Roux-en-Y Portoenterostomy	0	0	0	7	7	7.8%
Primary Repair over T-Tube	1	14	0	0	15	16.7%
Total	24	48	11	7	90	100%

**Table-III:** Post-Operative Morbidity (n=90).

Complications	No.	Percentage
Bile Leakage Through drain	8	8.9%
Wound Infections	15	16.7%
Recurrent Cholangitis	18	20.0%
Anastomotic Stenosis	3	3.3%

Four (4.4%) patients died due to uncontrolled sepsis, and multiorgan failure. These patients had undergone initial surgery at Afghanistan and presented late for definitive treatment. The hospital stay was 6-15 days. Patients were followed up for 6 months. Mortality rate was 4.4% and the success rate was 95.6%.

## DISCUSSION

In the current study, males were 16.7% and females 83.3% with male to female ratio of 1:5. Khan JS et al.^13^ reported13.8% males and 86.2% females (MF = 1:6.2). Shaikh R et al.^14^ reported male to female ratio of 3:4 in open cholecystectomy and 3:12 in laparoscopic cholecystectomy.

The main presenting features in this study, were pain in the right hypochondrium and epigastrium (77.8%), jaundice (76.7%), fever (74.4%), vomiting (64.4%), biloma (50.0%) and persistent biliary leak in 40.0% while 11.1% had developed biliary peritonitis. A local study reported abdominal pain, jaundice, fever and vomiting in 87.5% cases.^7^ Sohu KM et al.^5^ reported biliary colic (83%), dyspepsia (60%) and flatulence (60%). Sharma M et al.^4^ mentioned external biliary fistula (59.4%), abdominal pain (15.6%), jaundice (3.1%) and septicemia (9.4%). Nasa M et al.^15^ reported bile leak and biliary obstruction in his study. Bile leak presented in the form of diffuse abdominal pain (75.0%), nausea (62.0%), fever (77.0%), bloating ((61.0%) and anorexia (65.0%).

ERCP and MRCP are considered as the investigations of choice to reach a definitive diagnosis in cases of biliary injury.^12^ We performed abdominal ultrasonography in 81.1 % referred cases to identify collection and anatomy of biliary structures which is less than 90.6% and 100%^7^,^12^ but is almost comparable to 75% reported by Bakhsh R et al.^10^ Hela AH et al.^16^ reported single stone in 25.1% cases and multiple stones in 73.8% patients during pre-operative abdominal ultrasonography. Gall bladder polyps were demonstrated in 1.1% cases. ERCP (27.7%) and MRCP (81.1%) were performed in all those cases who presented late and had persistent complaints in spite of initial conservative management. These figures are comparable to 43.7% (ERCP) and 81.2%. (MRCP) mentioned in a local study^7^ but higher than those reported in other studies.^10^-^12^

In the current study, we had 26.7% CHD injuries, 53.3% CBD injuries and 12.2% patients had developed biliary Strictures. Seven (7.8%) patients sustained injury at the porta hepatis level. A local study has reported almost comparable figures.^7^ Sohu KM et al.^5^ reported 0.27% bile duct injuries. Khan JS and Ahmad R et al.^8^ reported 0.6% bile duct injuries in their study. Khan JS et al.^13^ demonstrated 0.85 biliary injuries in another study. Mostafa ESA et al.^6^ reported 45% cystic duct leak, 15% CHD leak and CBD leak. Schmidt SC et al.^17^ reported occlusion of CBD (9.0%), lateral lesions (26.0%), complete transection (44.0%) and late strictures (20.0%). According to Bismuth classification, 53.4% patients had Type-I injury, 26.6% had Type-II and 7.7% had Type-IV injury. Renz BW et al.^18^ classified the biliary injuries according to Stewart-Way classification. He reported class-I injuries in 5.0% cases, class-II in 20.0% cases, class- III in 35.0% cases and class-IV in 60.0% patients. Karanikas M et al.^19^ reported 61.0% patients sustaining Bismuth level 3, 4 and 5 injuries. Faridoon S et al.^20^ noted 12.5% Type-II injury and 12.5% sustained Type-III injury. Shaikh R et al.^14^ reported bile duct injuries according to Bismuth classification. He presented open and laparoscopic cholecystectomies separately. In open cases, Type-I injuries were 9.1%, Type-II were 18.2% and Type-III were 4.5%. In laparoscopic cholecystectomies, Type-I injuries were 13.6%, Type-II 22.7%, Type-III 18.1% and Type-IV injuries were 13.6%. Roux-en-Y hepaticojejunostomy is a definitive procedure for bile duct injuries and regarded as the gold standard.^10^,^12^,^14^,^17^

In the current series, 54.4% patients underwent Roux en-Y hepaticojejunostomy for injuries to CHD (n=23), CBD (n=20) and CBD strictures (n=6). This figure is comparable to 59.3% by Hadi A et al.^7^ Bakhsh R et al.^10^ reported 45% and Faridoon S et al.^20^ had 65% Roux-en-Y choledochojejunostomy reconstruction. Pesce A et al.^21^ recommended Roux-en-Y hepaticojejunostomy in cases of difficult end to end repair and sever biliary strictures. Other local and international studies have mentioned almost similar results.^12^,^14^,^22^

Viste A et al.^2^ reported 71.1% hepaticojejunostomy, Sharma M et al.^4^ had 43.75% Roux-en-Y hepaticojejunostomy, Mostafa ESA et al.^6^ had 7.5% choledochojejunostomy and Khan JS et al.^8^ coded 16.7% choledochojejunostomy. Choledochodudenostomy was performed in 21.2% cases which is comparable to 25% by Faridoon S et al.^20^ Roux-en-Y Portoenterostomy was performed in 7.8% cases for injuries at the level of porta hepatis which is comparable to 9.37% reported in a Local study.^7^

In 16.6% cases, intra operative injury was noted during the initial surgery which were managed by primary repair over a T-tube. This figure is less than 29.4% by Sawaya DE Jr^23^ but is comparable to 9.38% and 10% in other studies.^7^,^10^ Khan JS et al.^8^,^13^ reported 83.3% and 76.5% primary repair in his studies. Viste A et al.^2^ mentioned 8.8% cases of primary repair. The postoperative complications noted, are similar to those mentioned in literature. These include 8.8% cases of bile leak which is comparable to 6.25% mentioned in a local study.^7^ Al-Kubati WR et al.^11^ reported 9% bile leak in cases operated by senior surgeons and only 4.5% for Junior surgeons. Viste A et al.^2^ noted 9% bile leak following definitive surgery.

The incidence of wound infection was 16.6% which is less than 40.4% by Al-Kubati WR et al.^11^ Viste A et al.^2^ had 1.5%, Sohu KM et al.^5^ had 1.6% and Hadi A et al.^7^ reported 12.5% wound infection. Eighteen (20%) patients developed recurrent cholangitis with recurrent episodes of pain which needed hospitalization and were treated conservatively. This figure is higher than 10% by Shaikh R et al.^14^ but is comparable to 28.13% reported in a local study.^7^ Schmidt SC et al.^17^ reported 19.0% cases of post-operative cholangitis in his study of 54 cases.

In our study, 3.3% patients developed stenosis at the anastomotic site. One patient had undergone Roux-en-Y hepaticojejunostomy and other two patients had Primary repair of bile duct. These three patients underwent revision surgery. This morbidity is almost comparable to 4.54% and 6.25% mentioned different local studies.^7^,^14^ Viste A et al.^2^ reported 13.6% stricture formation following hepaticojejunostomy and 7.5% stricture formation after all reconstructive procedures. Khan JS et al^8^ recorded 0.13% CBD stricture formation following choledochojejunostomy. Mostafa ESA et al.^6^ reported 25% CHD stenosis and 10% CBD stenosis. Sharma M et al.^4^ noted 7.1% revision surgery for poor outcome following hepaticojejunostomy. The total hospital stay was 6-15 days. The success rate was 95.6%. It is comparable to 92.8% by Sharma M et al.^4^ The mortality rate was 4.4%. These four patients died due to uncontrolled sepsis because of their delayed referral from Afghanistan. The mortality rates mentioned in literature are 6%, 3.13%, 4.54%, 14.28% and 9.0% respectively.^2^,^7^,^12^,^14^,^17^ Schreuder AM et al.^24^ has reported zero mortality in his study.

### Strength of the study:

The prospective design is the strength of this study besides adequate sample size, classification of injury according to Bismuth classification, postoperative morbidity (Bile leak, stricture formation and improved quality of life) and comparison of reconstructive procedures employed in terms of better quality of life. The study also compares the different reconstructive procedures in terms of postoperative outcomes. Roux en Y hepaticojejunostomy was found to have excellent long-term results.

### Limitations:

Single center study, Different operating surgeons of surgical department performed these procedures. This could influence the outcome of procedures because of difference in their experience, Delayed presentation of patients after sustaining injuries, from remote areas and Afghanistan which increased the morbidity, hospital stay and mortality, Patients were reluctant for follow up visits especially belonging to remote areas. They were called on telephone calls.

### . Recommendations:

In future prospective multicenter studies with proper follow up for outcome reporting and proper training in laparoscopic techniques should be planned. Knowledge about the anatomical variations especially for inexperienced surgeons and early referral to a tertiary care hospital preferably Hepatobiliary surgeon should be ensured for better results.

Inexperienced surgeons should be aware of the anatomical variations which can result in iatrogenic bile duct injuries. This study also highlights the importance of early referral of patients sustaining iatrogenic bile duct injuries as it is associated with better postoperative outcome.

## CONCLUSION

Common bile duct was the most commonly injured site during Laparoscopic cholecystectomy followed by common hepatic duct, Roux-en-Y hepaticojejunostomy performed by an experienced surgeon, is the procedure of choice with excellent outcome Iatrogenic bile duct injury following laparoscopic cholecystectomy, is a serious complication. Therefore, early and timely reconstruction is of utmost importance for better postoperative outcome and improved quality of life. Proper timing to operate, appropriate surgical procedure and postoperative outcomes help in making a proper decision about surgical intervention

### Author’s Contributions:

**AH:** Conducted the study, prepared the manuscript, Data analysis and is also responsible and accountable for the accuracy or integrity of the work.

**IK & FOS:** Literature search**,** Data collection, Critical Review.

All authors have read and approved the final version of the manuscript.
